# Unfolding of Lignin Structure Using Size-Exclusion Fractionation

**DOI:** 10.3390/polym15193956

**Published:** 2023-09-30

**Authors:** Audrey LaVallie, Anastasia A. Andrianova, Joshua Schumaker, Sarah Reagen, Shelly Lu, Irina P. Smoliakova, Evguenii I. Kozliak, Alena Kubátová

**Affiliations:** 1 Department of Chemistry, University of North Dakota, 151 Cornell St., Mail Stop 9024, Grand Forks, ND 58202, USA; alavallie@nhsc.edu (A.L.); anastasia.andrianova@agilent.com (A.A.A.); josh.schumaker@hotmail.com (J.S.); sereagen@gmail.com (S.R.); shellylu01@gmail.com (S.L.); irina.smoliakova@und.edu (I.P.S.); 2 Nueta Hidatsa Sahnish College, 220 8th Ave. E, New Town, ND 58763, USA; 3 Agilent Technologies, 2850 Centerville Rd., Wilmington, DE 19808, USA; 4 SCIEX, 1201 Radio Rd., Redwood City, CA 94065, USA; 5 North Dakota Office of the Attorney General, Crime Laboratory Division, 2641 E Main Ave., Bismarck, ND 58501, USA

**Keywords:** indulin AT/alkali/softwood kraft lignin characterization, lignin fractionation, preparative size-exclusion chromatography, lignin narrow molecular weight fractions

## Abstract

The heterogeneous and recalcitrant structure of lignin hinders its practical application. Here, we describe how new approaches to lignin characterization can reveal structural details that could ultimately lead to its more efficient utilization. A suite of methods, which enabled mass balance closure, the evaluation of structural features, and an accurate molecular weight (MW) determination, were employed and revealed unexpected structural features of the five alkali lignin fractions obtained with preparative size-exclusion chromatography (SEC). A thermal carbon analysis (TCA) provided quantitative temperature profiles based on sequential carbon evolution, including the final oxidation of char. The TCA results, supported with thermal desorption/pyrolysis gas chromatography–mass spectrometry (TD-Py-GC-MS) and ^31^P NMR spectroscopy, revealed the unfolding of the lignin structure as a result of the SEC fractionation, due to the disruption of the interactions between the high- and low-MW components. The “unraveled” lignin revealed poorly accessible hydroxyl groups and showed an altered thermal behavior. The fractionated lignin produced significantly less char upon pyrolysis, 2 vs. 47%. It also featured a higher occurrence of low-MW thermal evolution products, particularly guaiacol carbonyls, and more than double the number of OH groups accessible for phosphitylation. These observations indicate pronounced alterations in the lignin intermolecular association following size-exclusion fractionation, which may be used for more efficient lignin processing in biorefineries.

## 1. Introduction

Lignin is one of the most abundant sources of renewable carbon: it builds up within the walls of plant cells and contributes up to 30% of biomass feedstocks [1,2]. As of 2019, the annual lignin market was estimated at USD 955 million with a projected 2% increase annually [3,4,5]. Due to its polyphenolic structure, a variety of value-added chemicals may be manufactured from lignin [6]; nonetheless, and despite significant efforts, the commercial use of lignin has not exceeded 2% of its annual production [3]. The disadvantage inherent to lignin is its irregular structure, which is composed of randomly cross-linked phenylpropanoid units [7]. An additional, and often underestimated, level of irregularity is caused by strong supramolecular aggregation, as lignin consists of multiple components having varied molecular weights (MW) [8,9,10,11]. The heterogeneous nature of lignin hinders a thorough characterization of its structure and, thus, limits its utilization [12]. The fractionation of lignin (desirably scalable) is considered to be a method whereby more homogenous subsections may be rendered: narrower molecular sizes and molecular weight (MW) distributions would greatly increase its homogeneity [5]. In addition, lignin fractionation is expected to break or reduce its aggregation, although this specific effect has not been documented.

Currently, three main methods have been used for lignin fractionation, either alone or in combination [13,14,15,16], i.e., selective solvent fractionation [5,12,17,18,19,20,21,22,23,24,25,26], differential precipitation [15,27,28,29,30,31,32,33,34], and membrane ultra- and nanofiltration [13,14,15,16,17,18,19,20,21,22,23,24,25,26,28,29,30,31,32,33,34,35,36,37,38,39,40,41,42,43]. A recent review also considered ionic liquid-assisted and enzyme-assisted fractionation; however, these new methods have not become mainstream protocols [44].

Selective solvent extraction is based on the differing partial solubility of lignin fractions in various solvents, which range greatly in their polarity and ability to solubilize lignin. Usually, lower-MW species are more soluble [5], so a sequential solvent application allows for obtaining fractions with increasing MW. Duval et al. provided a detailed analysis of the solvent parameters’ influence on the MW of the dissolved fraction, leading to a suitable fractionation protocol [19]. A major advantage of this method is the application of relatively inexpensive instrumentation and a moderately easy scale-up process. On the other hand, solubility depends on both the structural features and the MW [40]. Similar issues are encountered when the selective precipitation of dissolved lignin is performed. Furthermore, a non-desired additional chemical alteration may take place [45].

Membrane filtration enables the direct fractionation of black liquor and has the advantage of a controlled separation determined by the MW through the variation in the membrane pore size [40,41,42,43,46,47,48,49,50]. A combination of solvent extraction and membrane-assisted ultrafiltration yielded consistent results [51,52,53]. However, the membranes tend to become foul and the filtration process is not readily scalable to satisfy industrial needs [22].

As early as 1969, Kirk et al. considered the preparative size-exclusion chromatography (SEC) fractionation of lignin using gel permeation chromatography (GPC) as an effective approach for collecting lignin fractions solely based on molecular size [45]. An apparent advantage of this method is that the molecular size cut-offs can readily be controlled by varying the retention-time windows for fraction collection post-SEC processing. Furthermore, SEC is known to be a scalable technique [54].

An SEC application for obtaining lignin fractions with a desired MW, i.e., the separation of nineteen fractions with the number-average MW ranging from 340 to 1250 Da, was reported by Botaro et al. [55] using Acetosolv sugarcane lignin, which had been precipitated with water addition. Fourier transform infrared spectroscopy (FTIR) was used for the analysis of the functional groups, while an analytical SEC demonstrated the differences in the MW distribution for each of the collected fractions [55]. The preparative SEC enabled fractionation, often producing fractions with low polydispersity indices; however, it yielded a fairly low mass range of only up to 1250 Da. While the obtained low MW could be due to the specific feedstock, the fractionation was performed with hydroxypropylated cross-linked dextran as the SEC stationary phase, which might have yielded a separation that was not based exclusively on the MW [56]. We and others have previously shown that the undesired non-SEC interactions may skew the MW-based separation, and that this problem is amplified when a hydroxylated stationary phase is used [56].

The non-SEC interactions between lignin and the stationary phase in SEC, purportedly arising from the heteropolymeric nature of lignin and the variety of its functional groups, have been considered as possible sources of error [57,58]. However, in our previous study, we demonstrated that a highly cross-linked porous polystyrene/divinylbenzene matrix-based (PSDVB) stationary phase enabled lignin separation based solely on its MW [56]. The accuracy of the MW determination was confirmed using independent methods, including matrix-assisted laser desorption ionization with time-of-flight high-resolution mass spectrometry (MALDI-TOF HR MS) and direct infusion electrospray ionization (ESI) with TOF HR MS, which we also developed for lignin MW analysis [59,60].

In this study, capitalizing on the development of a suite of methods for lignin characterization, we set out to determine whether lignin’s chemical structure correlates with its MW. Following lignin fractionation using a preparative SEC, with a scaling-up of the previously validated conditions [56], a detailed structural characterization of the obtained fractions was performed. The hypothesis tested herein was that the fractions of different MWs may also be structurally different. We also postulated that the suite of analytical protocols used in this study, which included both thermal and spectroscopic methods, could provide insights into the lignin structure and reveal significant supramolecular interactions, which may contribute to lignin recalcitrance.

Of those, the TCA application was the significant innovation enabling accurate carbon quantification and providing specific thermal evolution profiles. We have successfully applied this method, in combination with TD-Py-GC-MS, analytical SEC, and ESI-TOF HR MS, to lignin analysis, observing the changes in its chemical structure while accurately closing the mass balance of carbon [61,62]. However, this study, for the first time, uses TCA to explain the changes in the polymer thermal properties observed due to the lignin components’ interactions, an outcome that was not anticipated when developing this method.

## 2. Materials and Methods

### 2.1. Materials and Reagents

Alkali lignin was purchased from Sigma-Aldrich (St. Louis, MO, USA). It was determined to have an elemental composition of C (64.14%), H (5.79%), S (1.39%), and N (0.46%) by Atlantic Microlab, Inc. (Norcross, GA, USA). HPLC grade unstabilized tetrahydrofuran (THF), containing no preservatives, was obtained from Fisher Scientific (Fair Lawn, NJ, USA). Deionized water was obtained using a Direct-Q^®^ 3 system from Millipore (Billerica, MA, USA).

For the preparative SEC, the alkali lignin was dissolved entirely in a 1:1 (*v*/*v*) THF/water mixture at a concentration of 50 mg/mL and further diluted with THF to form a lignin solution with a final concentration of 10 mg/mL, containing 10% of water. When the water content was decreased, no precipitation was observed.

For column calibration, two sets of narrow-range polymeric standards were used, i.e., a polystyrene (PS) standard set with a MW peak maxima (M_p_) of 580–19,760 Da, and polymethyl methacrylate (PMMA) standards (M_p_ 550–56,600 Da) from Agilent Technologies (Santa Clara, CA, USA). Pinoresinol (≥95% purity, Sigma Aldrich, Burlington, MA, USA) was used as the lignin structure model compound to verify the calibration, as suggested in the previous SEC validation study [56].

For the ^31^P NMR analysis, a set of lignin model compounds, including phenol, guaiacol, methyl guaiacol, ethyl guaiacol, propyl guaiacol, vanillin, acetovanillin, syringaldehyde, vanillic acid, homovanillic acid, and bicreosol (all ≥95% purity, Sigma Aldrich) were analyzed with respect to the chemical shift, for identification and quantitation. Pyridine (≥99.8%), cyclohexanol (≥99%), 2-chloro-4,4,5,5-tetramethyldioxaphospholane (TMDP) (≥95%), deuterochloroform (≥99.8%), and chromium acetylacetonate (≥97%) (all from Sigma Aldrich) were used for sample preparation.

### 2.2. Lignin Fractionation via Preparative SEC

To confirm that the SEC separation was primarily controlled by size exclusion, SEC calibration was performed with two sets of standards differing in their functional groups (PS, PMMA), as was demonstrated to be effective in our previous work (Figure S1 and Table S1) [56]. Additional details can be found elsewhere [60].

The preparative SEC fractionation was performed on an Agilent 1100 Series HPLC system utilizing a preparative PLgel column (300 × 25 mm, with 10 µm particle size and a 1000 Å pore size) (Agilent Technologies, St. Cloud, MN USA). The system was equipped with a diode array detector (DAD). For this work, the analytical flow cell was replaced by a preparative flow cell (Agilent Technologies). An unstabilized THF was used as the mobile phase at a flow rate of 5.0 mL/min; it was essential to use unstabilized THF to obtain pure lignin fractions without butylated hydroxytoluene or other additives used for THF stabilization. An extended loop capillary was installed in the injection loop to perform a 500 µL injection of a 10 mg/mL lignin solution.

Several fractionation trials (with the SEC chromatograms shown in Figure S2), which obtained 4–6 fractions, were performed by slightly varying the collection-time windows, yet provided comparable results (the SEC data from the preliminary fractionations are shown in the Supplementary Information). The final protocol targeted the collection of representative fractions with differing MW. For example, the pre-eluate (i.e., the fraction collected before fraction 1, which is henceforth labeled as that with the highest MW) was not further considered because it contained mere traces of organic carbon.

In the final fractionation protocol, the pre-eluate was collected first, during the retention times when no increase in the DAD signal was observed. Then, MW fractions 1–5 were obtained during the following elution-time windows: 14–16, 16–18, 18–20, 20–22, and 22–24 min. The fraction collection was performed manually. The procedure was repeated ten times, resulting in a final volume of 100 mL for each of the six collected fractions. Each fraction was concentrated using evaporation under a stream of nitrogen to a final volume of 2 mL. As the control, an aliquot of THF (100 mL) was also concentrated to a final volume of 2 mL.

### 2.3. Analytical SEC of Lignin Fractions

The obtained preparative SEC fractions, the control sample (concentrated THF), an aliquot of pure THF, and an untreated lignin solution (50 mg/mL) were analyzed using an analytical-scale SEC on an Agilent 1100 Series HPLC system equipped with a DAD with an analytical high-pressure flow cell, utilizing a PLgel analytical column (300 × 7.5 mm, with a 5 µm particle size and a 1000 Å pore size, 500–60,000 Da separation range) equipped with a PLgel guard column (50 × 7.5 mm) (Agilent Technologies). The SEC column with a PSDVB stationary phase was calibrated with PS standards (Figure S1). Unstabilized THF was used as the mobile phase at a flow rate of 1.0 mL/min. The injection volume for all samples was set to 20 µL.

The SEC analysis DAD abundance profile was based on the absorbance in a UV-Vis range of 220–700 nm. The SEC determination of the MW as *M_n_* (number-average MW), *M_w_* (mass-average MW), and dispersity index (DI) values were based on standard SEC equations [56].

Standard deviations are not provided in pertinent Tables and Figures because the results are reported for a single preparative SEC separation. The results were confirmed by four similar prior experiments (shown in the Supplementary Materials), with some of the fractions being collected on a slightly modified time scale, which prevented an exact statistical evaluation. Nevertheless, when the outcomes were compared, the variance did not exceed 10% for similar fractions.

### 2.4. Thermal Carbon Analysis

A thermal optical analyzer from Sunset Laboratory Inc. (Portland, OR, USA) was employed to obtain the quantitative thermal carbon evolution profiles (TCA), which enabled a comprehensive carbon fractionation and characterization [62,63]. TCA is a relatively novel method of accounting for all the carbon mass in an organic sample (converting all carbon to CO_2_ and then methane, with the uniform quantification of various carbonaceous species), with the added benefit that the temperature programs provide the fractionation by volatility, i.e., higher molecular weight compounds evolve at higher temperatures. Further, the evolved fractions are separated into two kinds: (1) those due to thermal physical desorption, from an ambient temperature up to 300 °C, and (2) pyrolytic products which evolve at higher temperatures, reflecting those lignin components that cannot volatilize without chemical decomposition—i.e., higher-MW compounds. The remaining material, recalcitrant char, is then evolved and recovered via combustion, thus closing the mass balance on carbon [62].

A sample (20 µL) was introduced into a Pall Flex 2500QAT-UP tissue quartz filter (Pall Corp, East Hills, NY, USA), dried on a hot plate at 40 °C for 4 min, and placed into the oven. The sample was desorbed/pyrolyzed at selected temperature steps for specific time durations. A detailed description of the applied TCA protocol can be found elsewhere [62,63]. Briefly, the thermal desorption temperatures were 30, 200, and 300 °C, while pyrolysis took place at 400, 500, and 890 °C in a helium atmosphere. This sequence was followed by oven-cooling to 550 °C, the introduction of an oxidizing carrier gas mixture of He with 10% O_2,_ and heating to 890 °C in order to evolve the coked carbon fraction. All the evolved species were converted to CO_2_ and then to methane, thus allowing for quantification using a single standard with a flame ionization detector [62].

### 2.5. ESI-TOF HR MS Analysis

The HR MS mass distribution of the different lignin fractions was obtained using an Agilent 6210 LC/TOF with a mass resolution of >13,000 (at *m*/*z* 2722) and mass accuracy <2 ppm (*m*/*z* 609.2807) with ESI [59]. The samples were introduced via direct infusion with a syringe pump at a flow rate of 5.0 μL/min. The analysis was performed in the positive ion mode with electrospray ionization (i.e., the capillary potential) and collision-induced dissociation (the fragmentor potential) set at 3500 and 150 V, respectively. Nitrogen, at a flow rate of 4 L/min, was used as the nebulizing gas. The nebulization temperature and pressure were set to 250 °C and 20 psi, respectively. The TOF MS system was calibrated with [(CsI)_n_ + Cs]^+^ clusters formed by an introduction of cesium iodide (30 mmol/L solution in ACN/water 1:1 (*v*/*v*)) via direct infusion at a flow rate of 5 μL/min.

The Mass Hunter software package B.07.00 (Agilent Technologies, St. Cloud, MN, USA was used for data processing. The mass spectra of lignin were deconvoluted using a built-in tool utilizing an unbiased isotope model with a peak spacing tolerance of 0.0025 *m*/*z*. The maximal assigned charge state was not limited. Hydrogen (proton) was considered as the charge carrier. The peaks selected for deconvolution were filtered based on their absolute height (≥100 counts) and the relative height of the largest peak, which was set to ≥0.1% of the largest peak unless otherwise stated. The maximum number of peaks was not specified.

### 2.6. TD-Py-GC-MS Analysis

TD-Py-GC-MS was performed on a CDS Analytical Inc. 5200 pyroprobe (Oxford, PA, USA), connected to an Agilent GC 7890 with 5975C MS. The quartz tube with quartz wool was cleaned outside of the probe at 1200 °C for 5 s. The sample was introduced at a 5.0–10.0 µL volume onto the quartz wool filter before the probe was inserted and, once inserted, the probe was heated sequentially through 200, 300, 400, 500, and 890 °C steps, just as in our original publication [62]. The probe was held at each temperature for 30 s, except at the 890 °C step, which was held for 10 s. The transfer line and valve oven were kept at 300 and 320 °C, respectively, and the pyroprobe assembly was held at 300 °C. The temperature steps of 200, 300, 400, 500, and 890 °C were repeated twice during the runs to ensure that all potential polymers had evaporated for the online GC-MS analysis. Additional details of this experimental setup are provided elsewhere [62,63].

The GC-MS was equipped with a 51 m HP 5MS column (0.25 µm film thickness and 0.25 mm inner diameter). The GC inlet was kept at 300 °C, with a 10:1 split ratio. The GC oven temperature program started at a 50 °C hold for 1 min, followed by a 40 °C/min gradient up to 80 °C, and then used the second gradient of 25 °C/min, up to 320 °C, with a final hold of 7 min. The MS analysis was performed with no solvent delay in the 10–550 *m*/*z* mass range. The resulting GC-MS data showed that the second run for each pyroprobe temperature yielded no residual species before the next increased temperature step, hence, indicating no carryover. The total ion current (TIC) chromatograms of the fractions and blank sample were analyzed for lignin-derived compounds; the peaks were labeled when the compounds were identified with ≥80% accuracy for the NIST library search results or based on confirmation with the standards [62].

### 2.7. ^31^P NMR Analysis

A Bruker AVANCE 500 NMR spectrometer was used to record the ^31^P spectra. Samples for the proton-decoupled ^31^P NMR (to be called just ^31^P NMR henceforth), were obtained using a mixture of pyridine and CDCl_3_ (ratio of 1.6:1). For the quantitative ^31^P NMR studies, the pulse width was optimized to give the 90° flip angle at approximately 10 µs. The optimized pulse delay was 20 s. The ^31^P NMR spectra of the TMDP and its hydrolysis product were obtained at 256 scans, while the spectra of the phosphitylated lignin, lignin degradation products, and other analytes were obtained using 1024 scans.

The general procedure for the phosphitylation reaction was as follows, using a combination of the published protocols [64,65]: A mixture of pyridine and CDCl_3_ (400 µL of 1.6:1 (*v*/*v*)) was added to a 4.0 mL vial with a magnetic stir bar. Then, a sample to be phosphitylated was introduced into the vial. During the quantification studies, chromium acetylacetonate (1.0 mg) and cyclohexanol (10 µL, the internal standard for integration) were added to the vial before introducing the phosphitylation reagent. Two molar equivalents of TMDP were added dropwise to the solution. After stirring at room temperature for 5 min, the phosphitylated sample was transferred to an NMR tube. The ^31^P NMR spectra were recorded within 1 h after preparation of the sample. Prior to phosphitylation, the samples that were dissolved in the water/DCM were dried using a rotary evaporator at 20 torr for 60 s to remove the solvents from the system, since the presence of hydroxyl groups in the solvents was not conducive to the phosphitylation reaction.

The ^31^P NMR signal of the phosphitylated cyclohexanol was observed at δ 145.2 ppm. A sample of hydroxylated TMDP, 2-hydroxy-4,4,5,5-tetramethyl-1,3,2-dioxaphospholane, was produced by adding two droplets of water to a solution of 250 µL pyridine, 150 µL CDCl_3_, and 15 µL TMDP, which was stirred for 5 min. The ^31^P NMR signal of the hydroxylated TMDP was observed at δ 132.2 ppm.

The number of hydroxyl groups present in each sample was calculated through integration relative to the cyclohexanol peak found at 145.2 ppm with a known concentration. The following regions were used for the classification of the hydroxyl groups detected in the weight-fractionated samples: 143–150 ppm for alcohols, 138–143 ppm for phenols, and 135–138 ppm for carboxylic acids.

Prior to conducting the NMR studies of the fractions, we verified the method used by matching its results to the amounts of the hydroxyl groups in indulin reported by Meadwest Vaco in the manufacturer’s certificate of analysis (Table 1). It is of note that the phosphitylated benzylic and aliphatic hydroxyl groups are indistinguishable using ^31^P NMR spectroscopy, and the number of aliphatic moieties given in Table 1 includes the hydroxyl groups at the benzylic position. While the number of aliphatic and, especially, phenolic hydroxyl groups determined using ^31^P NMR spectroscopy is somewhat lower, the difference between our data and those of Meadwest Vaco for the total amount of OH-containing groups was negligible when the carboxylic acids (not reported by Meadwest Vaco) were added.

## 3. Results

**Figure 1:**
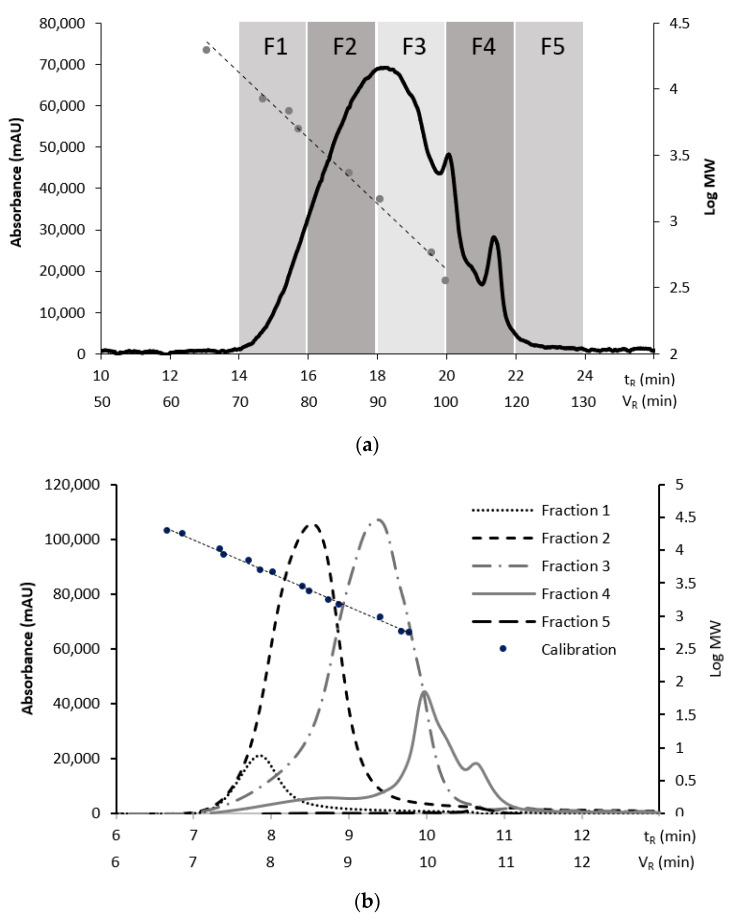
SEC analysis of (**a**) chromatogram denoting alkali lignin fractionation using preparative SEC showing the collection of five fractions (see experimental section for detail) and (**b**) chromatograms of fractions analyzed with analytical SEC. The dotted straight line with data points, as well as the right vertical axes, refer to the SEC calibration. (**a**) Preparative SEC and (**b**) analytical SEC.

As demonstrated in our previous work, the application of a highly cross-linked porous PSDVB stationary phase enabled lignin separation, which was truly based on MW, according to the calibration using both polar and non-polar standards [56]. So, in this study, lignin was effectively separated into five main fractions via preparative SEC (Figure 1). As a result, a narrower MW distribution of the species within the fractions, as compared to the original, intact lignin, was achieved.

### 3.1. Mass and MW Distribution in SEC Fractions

The distribution of lignin among the fractions obtained using the preparative SEC (Figure 1a) was assessed using two methods based on (i) the TCA results enabling the accurate quantification of carbon and (ii) an analytical SEC with UV/Vis detection (Figure 1). Similar distribution profiles were obtained using both methods (Table 2), suggesting that the UV/Vis response was mostly based on the contribution of lignin aromatic moieties, which made up the bulk of the lignin mass. It is of note that the TCA provided the inherently non-biased carbon abundance, as all the carbon was evolved, and then all of the evolved carbon was quantified as a single analyte, methane. Thus, the fraction abundance obtained using TCA presents the true values, which are absolute rather than relative. Given this feature, the obtained TCA data are re-visited in Section 3.4, where they are matched to the TD-Py-GC-MS profiles.

The general trends of the analytical SEC based on the average MW values, *M_p_*, *M_n,_* and *M_w_* listed in Table 3, were as expected, showing sequentially decreasing values. Large-MW species eluted first, and the following fractions featured successively lower-MW species, presumably phenolic oligomers, then dimers with monomers.

However, a closer look at the analytical SEC profiles acquired for each fraction revealed rather broad peaks of a non-Gaussian shape (Figure 1b) for each fraction, indicating a possible carryover of high-MW lignin components into the late fractions, as well as the occurrence of low-MW components in the early fractions.

This observation indicated strong interactions between the unfractionated lignin components of varied MWs within the original, intact lignin structure. We hypothesized that lignin fractionation broke some of these interactions, leading to a partial “unraveling” of the aggregated lignin structure. Evidence of the supramolecular structure of lignin was reported earlier [10,11]. As an extreme case, Crestini et al. showed that milled wood lignin components are associated so strongly that the resulting polymer was actually an aggregate of linear oligomers [9]. The rest of this study reports and discusses the evidence confirming the correctness of this assumption obtained using various analytical methods.

The comparison of the expected MW (obtained using the preparative SEC) and the actual *M_n_*, *M_w_*, and PI (based on the analytical SEC) presented in Table 3 supports the occurrence of intermolecular interactions between the unfractionated lignin components of varied MWs and their disruption as a result of their run through the preparative and then the analytical SEC columns. Presumably, these aggregates partially disassembled and became separated from each other. The contributions of the species with higher-than-expected MWs were rather sizable, particularly for fractions 4 and 5 (Table 3). Note that while fraction 5 is small in abundance, the obvious contribution of the higher-MW species cannot be ignored.

Interactions between lignin components have been observed earlier; the interest in studying them stems from the fact that they are responsible for the formation and high stability of lignin nanoparticles [10,11]. The tendency toward aggregation and particle size have been shown to depend on the extracting solvent [12,66], suggesting some dependence on the MW. However, to the best of our knowledge, no direct evidence of the particular interactions between low- and high-MW lignin components with defined MWs has been reported until this study.

The observed peak broadening (in Figure 1) is unlikely to have been caused by common chromatographic artifacts, such as peak fronting, caused by the overloading of separation sites on the preparative SEC. This phenomenon was not observed in our earlier study [56], nor did it become apparent in the first, preparatory SEC run in this study, even though the column loading was significantly higher. Similarly, column interactions, besides size exclusion, which could potentially cause peak tailing were not observed during the first SEC run.

However, verification using independent methods was still warranted. The average MW values obtained with SEC calibration were verified using a complementary method, ESI-TOF HR MS (developed earlier) [59], as shown in the next section. Then, corroboration was sought for the occurrence of interactions between the high- and low-MW lignin components by comparing the thermal behavior of the SEC fractions and native lignin using two independent methods, TCA and TD-Py-GC-MS.

### 3.2. ESI-TOF HR MS Analysis of SEC Fractions

**Figure 2:**
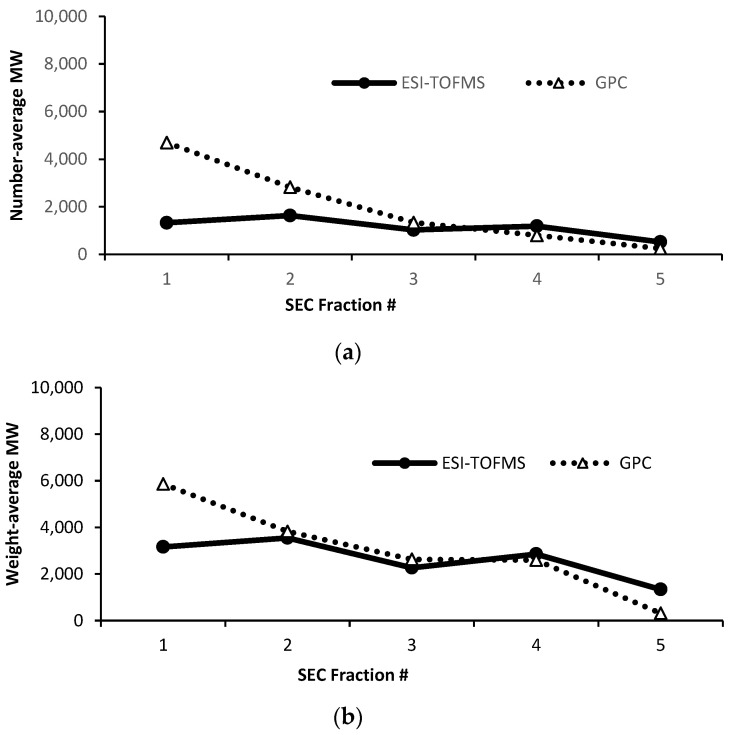
Comparison of SEC vs. ESI-TOF HR MS number-average and weight-average molecular weights for all lignin fractions. (**a**) *M_n_* and (**b**) *M_w_*.

The ESI-TOF HR MS, just as the SEC did, showed sequentially smaller number-average and weight-average molecular weights (*M_n_* and *M_w_*) values as the fractions proceeded from 1 to 5, while the unfractionated lignin showed *M_n_* and *M_w_* values in the mid-range of the fractions’ values (Figure 2, Table S2). The actual ESI-TOF HR MS profiles of fractions 1–5, shown in Figure S3, also reflect this expected trend through the sequence of the mass spectra: fraction 1 clearly shows an abundance of mid- and high-MW values for the compounds, while fractions 2–4 feature steadily fewer high-MW peaks and then fewer mid-MW species and, finally, by fraction 5, only low-MW compounds were present in the amounts detectable using MS.

The analytical SEC and ESI-TOF HR MS yielded similar, average-MW values for fractions 2–4, which comprise the bulk of the fractionated lignin. The difference between the values obtained using MS and SEC was pronounced only for fraction 1, which is consistent with the limitation of MW determined using MS, as large-MW species may not volatilize and may excessively fragment in comparison to lower-MW species. It is of note that the low abundance or even apparent absence of high-MW peaks in fractions 3–5 does not necessarily mean that they are absent: unlike the UV detection used in the SEC, the HR MS signals of those species present below their detection limits do not accumulate.

Thus, the carryover of the high- and low-MW species observed in the SEC could not be confirmed by the ESI-TOF HR MS due to this method’s limitations. Confirmation of this was obtained by using thermal methods, as shown in the next section.

### 3.3. Quantitative Profiles of SEC Fractions

To explain the observed broad-peak profiles suggesting interactions between the low- and high-MW species (Figure 1b and Table 3), fractions 1–5 were subjected to a further detailed quantitative and qualitative chemical characterization. A TCA was used for quantification, while a TD-Py-GC-MS and ^31^P NMR provided the structural characterization.

The TCA, in addition to determining the total carbon amount in each fraction (which was used for the verification of fraction abundance, shown in Table 2), provided the carbon distribution among the portions which evolved at different temperatures. As mentioned in Section 2.4, those were: (1) the TD directly evolved via evaporation, below 300 °C, (2) the products of the subsequent higher-temperature pyrolysis and, finally, (3) the char or coke, i.e., the material that was not broken down pyrolytically, even at 890 °C, which subsequently combusted in the presence of oxygen. Accounting for all the types of carbon enabled a comprehensive sample quantification.

**Figure 3:**
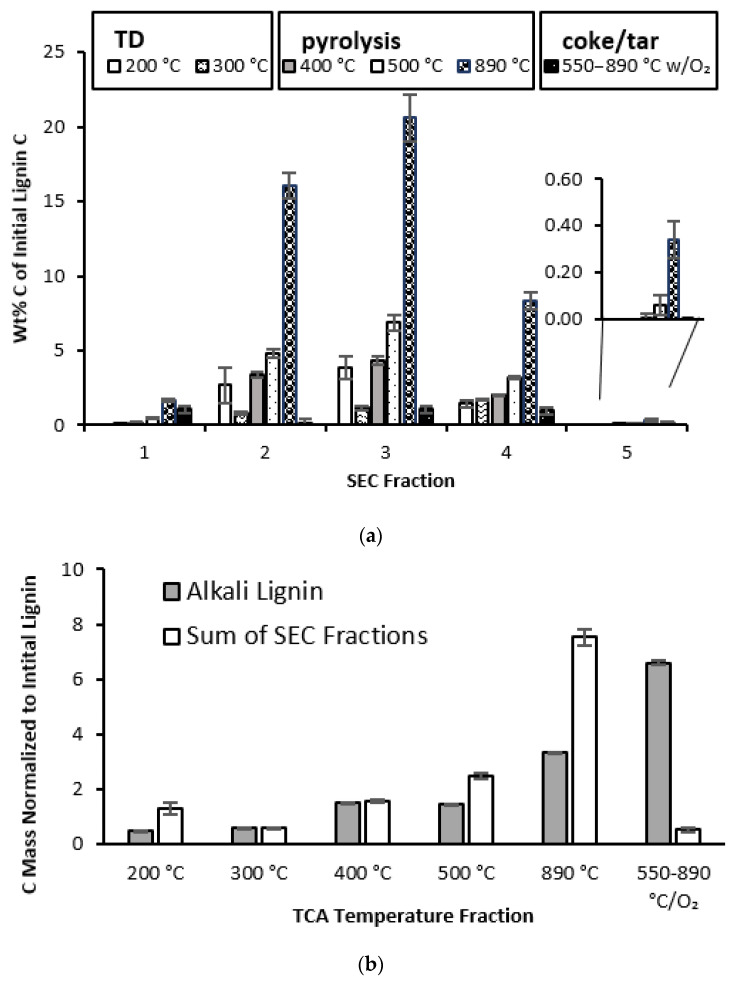
TCA analysis: (**a**) Carbon % distribution among the TCA temperature portions for each lignin fraction; the insert in panel (**a**) shows magnified fraction 5. (**b**) Carbon mass sums for all SEC fractions at each temperature step of TCA compared to the corresponding alkali lignin carbon masses.

Fractions 1–3 showed pronounced pyrolysis at 890 °C, which was expected due to their relatively high MW (Figure 3a). Less expected was the occurrence of sizable TD portions in their TCA profiles. The TCA thus confirmed that these SEC fractions contained some low-MW compounds which volatilized at TD temperatures, which was the observation made when using the analytical SEC. As mentioned earlier, the occurrence of species with unexpected MWs is possibly due to the strong adsorption of high- and low-MW compounds on each other, i.e., an aggregation specific to the mismatch in size of the interacting species.

The TCA profiles of fractions 4 and 5 corroborate this conclusion. It is of note that while fraction 4 is abundant, fraction 5 is not, so its magnified profile is shown in Figure 3a as an insert. Contrary to the expectation of their increasing volatilization at TD temperatures, their TCA profiles actually showed rather large portions which evolved at pyrolytic temperatures. This observation is consistent with the high-MW lignin components’ carryover into these fractions, as shown in Figure 1b.

Some high-MW chemicals may be formed as a result of heating, as the presence of adsorbed high-MW compounds may enhance polymerization. This phenomenon of the re-polymerization of low-MW lignin components during thermal treatment is well known [67,68,69,70]. Thus far, various methods, including TCA, have been used merely to confirm the MW profiles obtained using SEC. However, TCA, besides closing the mass balance on carbon, provides additional opportunities for revealing the structural changes occurring in lignin as a result of its fractionation by MW, particularly in combination with TD-Py-GC-MS.

Namely, the modes of thermal evolution were rather different for the fractionated lignin and its unfractionated precursor. The latter, unlike the former, appeared to have stronger interactions, most likely via hydrogen bonding, creating a network similar to cross-linking. This intermolecular cross-linking may hinder the pyrolytic evolution of small-MW products, i.e., causing polymerization/charring. This is apparent when comparing the TCA profile of intact lignin to that of the sum of all the fractions in Figure 3b, where the unfractionated lignin yielded about 47% char (coke), while 24% of the carbon was volatilized at 890 °C. In contrast, the amount of carbon which evolved at 890 °C without oxygen increased in the fractionated lignin, at the expense of the final carbon portion, which evolved only with oxygen.

This striking difference indicates that unfractionated lignin has a much greater propensity to charring, compared to any of its MW fractions, including even that with the highest MW. Apparently, charring is enhanced when polymers interact with abundant and accessible small-MW fragments, as is the case in the unfractionated lignin—this is a novel conclusion resulting from the current work. Presumably, the lignin molecules fold with the hydrogen bonds serving as a “glue”, with the smaller-MW components “cementing” the fold. Even though such interactions still skew the expected TCA pattern in fractions 4 and 5, the extent of their charring is nowhere near that of the original lignin, presumably because such interactions are much stronger in the latter.

These results are in agreement with the report of Chua et al., who made a crude separation by lignin dissolution in THF. They observed that both the THF-soluble and -insoluble fractions, presumably of low and high MWs, respectively, produced significantly less char upon pyrolysis than the original lignin, i.e., similar to what we observed for all MW fractions [71].

These results are also consistent with the study of Li et al., who reported that magnesium and calcium hydroxides tend to reduce lignin agglomeration, i.e., the interactions between the lignin components. As a result, more organic phenolic compounds and CO_2_ were formed upon the pyrolysis of this material with reduced interactions, at the expense of the hard-shelled char characteristic of the untreated lignin [72].

The next question was what type and size of compounds volatized at each temperature step, and whether those profiles were different for the fractionated and native lignin. This relationship was investigated using the TD-Py-GC-MS speciation data.

### 3.4. TD-Py-GC-MS: Speciation of Fractionated Lignin

**Figure 4:**
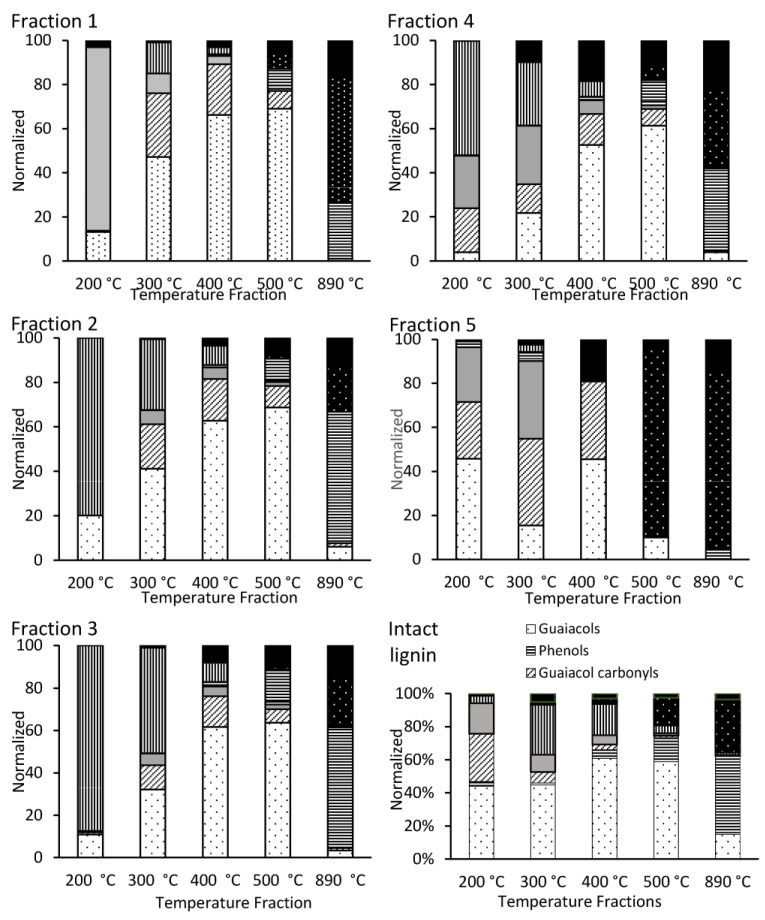
Normalized distribution of different classes of organic compounds for each TD-Py-GC-MS temperature for SEC fractions 1–5 and unfractionated lignin.

The five SEC fractions were analyzed via TD-Py-GC-MS to identify the various compounds which evolved sequentially at increasing temperatures. The corresponding profiles, including the non-fractionated lignin, are shown in Figure 4 (supported by Figures S4 and S5).

The semi-quantitative TD-Py-GC-MS of fractions 1–5 corroborated the TCA results, showing a similar total abundance as well as the premature elution of some low-MW compounds in the early fractions (1–3), and high-MW lignin components in the later fractions, 4 and 5 (Figure S4).

The identified volatile compounds (i.e., those thermally desorbed up to 300 °C) in the fractionated lignin consisted mainly of guaiacyl dimers, guaiacol carbonyls and acids, and guaiacols. In contrast, guaiacols as the homology series were the most abundant class of compounds in the early pyrolytic fractions (400 and 500 °C), followed by guaiacyl carbonyls and dimers. These observations were consistent with those observed earlier [61,62]. The general trends and mechanistic considerations can be found elsewhere [61]. Here, the focus will be on the differences between the SEC fractions.

With a further increase in temperature, other pyrolytic products—phenols, aromatic hydrocarbons and, ultimately, polycyclic aromatics (mostly retene and phenanthrenes)— appeared as immediate structural char precursors that became most abundant in the final, 890 °C temperature step. Approaching this temperature, the aromatic and polycyclic hydrocarbons became dominant in fraction 5, although they were abundant in all the fractions. These results are in agreement with those of Shao et al., who observed a more abundant formation of phenols and aromatic hydrocarbons upon the high-temperature (800 °C) pyrolysis of high-MW lignin components [73]. On the other hand, they are also consistent with the observation of Li et al., that polycyclic hydrocarbons tend to form as a result of the pyrolysis of low-MW lignin components [74].

The observation of abundant polycyclic aromatics in fraction 5 is not a common feature, though, because low-MW chemicals characteristic of this fraction are expected to volatilize rather than undergo pyrolysis. Thus, the observed “hard” pyrolysis is evidence of the presence of high-MW components. The fact that PAH formation is actually the most pronounced in fraction 5 (even more than in the unfractionated lignin) indicates that the formation of char precursors is most likely when a mixture of high- and low-MW components is pyrolyzed, suggesting their interaction.

The TD-Py profiles in the SEC fractions appear to be additive and, thus, qualitatively similar when compared to the native lignin (Figure 4). Thus, the SEC fractions are similar in their basic chemical structure, including the original lignin, corroborating a similar conclusion made in the literature for a larger-scale fast pyrolysis of lignin [73]. Nonetheless, some sizable differences, both quantitative and even qualitative, were observed, particularly in the TD fractions. The acetic and propionic acids which evolve at 200 °C were surprisingly abundant in fraction 1 and nowhere else, suggesting their preferential adsorption in high-MW species, presumably via hydrogen bonding. The other qualitative difference was the abundance of guaiacyl acids (predominantly homovanillic) and dimers in fraction 4 mentioned earlier.

In comparison to the original lignin, the volatile guaiacol carbonyls which evolve at TD temperatures were in a much higher abundance in the SEC fractions, the effect of which was also most pronounced in fraction 4. This difference may be explained by the stronger adsorption of these compounds (perhaps, with multiple hydrogen bonding) within the non-fractionated lignin, hindering their evolution, with a partial cleavage of these intermolecular bonds as a result of fractionation by MW. This explanation is consistent with the hypothesis of strong interactions between the lignin components of different MWs. For further confirmation of this hypothesis, and to reveal the chemical mechanism of such interactions, another independent method was used, ^31^P NMR, for the detection of hydroxyl groups potentially involved in hydrogen bonds formation.

### 3.5. Hydroxyl Group Quantitation using ^31^P NMR Spectroscopy

The ^31^P NMR spectra of the SEC fractionated lignin samples enabled the determination of the main functional groups (Figures S6 and S7). The number of mmoles found in each sample are summarized in Table 4. Then, the number of moles of the post-SEC hydroxyl groups was determined for all five fractions. Surprisingly, their sum turned out to be significantly larger (>2 times) than the number determined for the alkali lignin before fractionation.

It is noteworthy that the spectra of all the fractions also contained a cluster of unidentified signals of low intensity centered around 150.6 ppm. These signals (labeled as “unidentified” henceforth) have not been reported in other studies of phosphitylated lignin-based samples, nor were they observed in the spectra of the other lignin and lignin-derived samples investigated by our group. It is possible that the TMDP reacted with some other highly electronegative species such as sulfur, which is present in alkali lignin.

The most plausible explanation is that some hydroxyl groups in the unfractionated lignin before SEC were inaccessible for phosphitylation, due to steric hindrance. This observation corroborates the TCA results, which showed that the unfractionated lignin is much more recalcitrant toward pyrolysis, presumably due to the lignin fragments’ folding. The fractionation process possibly caused modifications of the complex three-dimensional structure of lignin in such a way as to allow more hydroxyl groups to undergo phosphitylation. This explanation is consistent with the observed, strong interactions between the low- and high-MW lignin fractions that may be responsible for lignin association. Li et al. observed that lignin fractionated by partial dissolution in acetone significantly increased the adsorption capacity toward a cationic dye, methylene blue [75], thus corroborating our findings.

It is of note that all the fractions made a sizable contribution to the observed increase in the number of phosphitylation-accessible OH groups, as compared to the original lignin. The contribution of the middle fractions was the greatest in terms of the absolute numbers. For example, the single fraction 3 showed an amount similar to that of the whole unfractionated lignin, with fractions 2 and 4 also making an impact.

Nonetheless, fractions 1 and 5 stood out in terms of their relative contribution to the OH group pool. Based on Table 4 (the rightmost column), fractions 1 and 5 contain sizable percentages of the hydroxyl groups, 11.2% and 8.8%, respectively. However, according to Table 2, these two fractions account for only 3.9 and 0.7% of the total carbon (determined using TCA), respectively. Thus, they, and particularly fraction 5, with the lowest MW, contain disproportionally large hydroxyl group contents (mostly at the expense of the most abundant fraction 3). Furthermore, fractions 1 and, in particular, 5, showed an enrichment in aliphatic hydroxyl groups at the expense of phenolic ones (Table 4). For the reader’s convenience, the relative distributions of the different types of hydroxyl groups are shown explicitly in Figure S6. The enrichment of fraction 5 with aliphatic OH groups contradicts the earlier observed enrichment of lower-MW fractions with phenolic OH groups [39,53]. However, these earlier data were obtained when using a different MW fractionation method, ultrafiltration. We also observed the enrichment with phenolic OH groups up to fraction 4—but not in fraction 5 (Table 4, Figure S6). This exception can be readily explained by the carryover of some fraction 1 material into it. Note that these two fractions, though of low abundance, contain the species with the greatest mismatch in size, i.e., those expected to form the strongest interactions prior to SEC fractionation.

These findings may explain the observed strong interactions between the low- and high-MW lignin components, resulting in the significant broadening of the SEC peaks (Figure 1b). They may also explain the difference in the composition of the TD fractions discussed in the previous section. The aforementioned interactions may be enabled by strong hydrogen bonding, which would reduce the effective charges. It is well known that lignin aggregation is more pronounced in non-hydrogen-bonding polar solvents, e.g., THF, where the solvent does not interfere with such interactions [9,10]. In turn, unfractionated lignin dissolution in a polar solvent may cause the transfer of such interacting hydroxyl groups into the internal domains of lignin-agglomerated particles, making them less accessible and less amenable to analysis.

Alternative explanations were also considered for the observed increased amount of OH groups in the fractionated lignin. Theoretically, the THF used in the SEC method could be a source of some of the additional OH groups identified in our experiments. For example, THF could have reacted with lignin under the conditions used during the SEC analysis, resulting in the cleavage of the THF ring which allowed for the formation of a new hydroxyl group. THF peroxide formation was also a possibility, as the product of this reaction had a new OH group and, therefore, would readily react with the TMDP. Finally, the ether bond cleavage in the THF could occur either before or during the SEC application. However, these qualitative considerations did not pass the quantitative test. Namely, the ^31^P NMR spectrum of the phosphitylated-concentrated THF contained two phospholane signals. The first was a signal in the aliphatic hydroxyl group region at 147.4 ppm, whereas the other was located in the phenolic region at 142.0 ppm. However, both signals had relatively small integrations and an insignificant impact on the final total. Furthermore, to eliminate any potential artifacts, the pre-eluate was used as a blank—and even after this treatment the sum of the MW fractions still yielded a greater amount of OH groups than the non-fractionated lignin (Table 4).

Thus, the unraveling of the lignin supramolecular structure appears to be the most plausible explanation of the observed mismatch in the OH group quantitation between the original lignin and the sum of its MW fractions, and is supported by their altered thermal behavior.

## 4. Conclusions

Lignin fractionation by MW using SEC showed strong interactions between its low- and high-MW components. The occurrence of these interactions was observed after the re-run of the separated MW fractions through the same SEC column, resulting in pronounced peak broadening. Such interactions were confirmed using several methods, including ESI-TOF HR MS, TCA, and TD-Py-GC-MS. The application of these methods, particularly TCA, for lignin MW fraction analysis was fruitful, having shown that fractionation by MW caused several significant and unexpected changes in lignin properties. In particular, the fractionated lignin showed a smaller propensity to charring as compared to the original lignin, as shown by the combination of TCA and TD-Py-GC-MS. Furthermore, the component structure was shown to be different among the obtained MW fractions, with an uneven distribution of phenolic and aliphatic hydroxyl groups. Namely, the number of accessible hydroxyl groups, e.g., those amenable to ^31^P NMR analysis, increased more than two-fold. As an apparent result, several volatile compounds, in particularl guaiacol carbonyls, were much more abundant in the TD fractions of the TD-Py-GC-MS of the fractionated lignin, at the expense of charring. These phenomena can be explained by the association between high- and low-MW lignin components in unfractionated lignin, presumably via hydrogen bonding, thus making a stable and recalcitrant particle that folds upon itself, forming supramolecular particles. After the SEC separation, lignin appears to unravel/unfold due to a partial loss of such an association, resulting in a reduction in its structural integrity. The observed reduction in lignin recalcitrance by fractionation may be used for its more efficient processing in biorefineries.

## Figures and Tables

**Table 1:** Comparison of the number of mmoles of hydroxyl groups present per g of alkali lignin reported by Meadwest Vaco (for indulin) vs. the results obtained in the present study using ^31^P NMR spectroscopy for alkali lignin and indulin.

		^31^P NMR Data	
	Meadwest Vaco Data	Alkali Lignin	Indulin
Phenolic OH	3.6	2.85 ± 0.04	2.4 ± 0.3
Benzylic OH	0.06	2.7 ± 0.2	2.4 ± 0.3
Aliphatic OH	2.9		
Carboxylic Acid OH	Not Reported	0.78 ± 0.01	0.7 ± 0.1
Total	6.5	6.3 ± 0.2	5.5 ± 0.6

**Table 2:** Distribution of lignin sample across fractions as measured using TCA (the data are proportional to mass) and DAD (based on UV-Vis absorption) following the preparative SEC.

Fraction #	1	2	3	4	5
**TCA % distribution of lignin across fractions**	3.9	32	43	20	0.47
**DAD % distribution of lignin across fractions**	5.6	37	43	14	0.30

**Table 3:** MW characterization using the analytical SEC of both the unfractionated alkali lignin and the five fractions obtained using the preparative SEC.

Analytical SEC						
Fractions	1	2	3	4	5	Alkali Lignin
*M_n_*	4698	2824	1342	802	248	1631
*M_w_*	5862	3823	2626	2595	313	2740
*M_z_*	6647	4943	4979	4932	396	3723
DI	1.2	1.4	2.0	3.2	1.3	1.7
Estimates based on preparative SEC						
Expected MW range (g/mol)	4300–13,300	1400–4300	440–1400	140–440	<140	
% species: in the expected range	58	69	61	50	5	
of MW > than expected	0	15	29	44	95	
of MW < than expected	41	15	10	5	0	

**Table 4:** Number of mmoles determined in NMR samples in the SEC weight fractions per g of alkali lignin.

	Alcohols	Phenols	Acids	Unidentified	Total
Fraction 1	0.62	0.35	0.00	0.02	0.99
Fraction 2	2.11	1.73	0.12	0.24	4.19
Fraction 3	2.81	2.03	0.20	0.09	5.13
Fraction 4	1.50	1.77	0.33	0.30	3.90
Fraction 5	0.31	0.08	0.04	0.00	0.43
Total of fractions	7.35	5.96	0.69	0.65	14.65
Intact lignin	2.67	2.85	0.78	ND	6.29

## Data Availability

Additional data supporting the reported results can be found in the Supplementary Materials.

## Supplementary Materials

The following supporting information can be downloaded at: https://www.mdpi.com/article/10.3390/polym15193956/s1, Table S1—Calibration data for PS and PMMA standards obtained for analytical SEC. Table S2—MW characterization of the five fractions obtained using preparative SEC and unfractionated alkali lignin conducted with ESI-TOF HR MS. Figure S1—Calibration curve for PS and PMMA standards obtained for analytical SEC. Figure S2—SEC preparative and analytical chromatograms for preliminary fractionation experiment. Figure S3—ESI-TOF HR MS spectra for SEC lignin fractions 1–5. Figure S4—Aggregated TD-Py-GC-MS peak areas based on the GC-MS TIC response. Figure S5—TD-Py-GC-MS profiles for each SEC fraction based on the TIC peak-areas response. Figure S6—^31^P NMR spectra of SEC weight-fractionated lignin samples. Figure S7—Relative abundance of characteristic functional groups determined using ^31^P NMR.
