# Supplementary material for: Unfolding of Lignin Structure Using Size-Exclusion Fractionation
Source: Polymers (Basel). 2023 Sep 30;15(19):3956. doi: 10.3390/polym15193956 (PMC10574856; doi:10.3390/polym15193956)

**Supplementary Materials**

# Unfolding of Lignin Structure Using Size-Exclusion Fractionation

**Audrey LaVallie [1,2], Anastasia A. Andrianova [1,3], Joshua Schumaker [1,4], Sarah Reagen [1,5], Shelly Lu [1], Irina P. Smoliakova [1], Evguenii I. Kozliak [1,*] and Alena Kubátová [1,*]**

1 Department of Chemistry, University of North Dakota, 151 Cornell St., Mail Stop 9024, Grand Forks, ND 58202, USA; alavallie@nhsc.edu (A.L.); anastasia.andrianova@agilent.com (A.A.A.); josh.schumaker@hotmail.com (J.S.); sereagen@gmail.com (S.R.); shellylu01@gmail.com (S.L.); irina.smoliakova@und.edu (I.P.S.)
2 Nueta Hidatsa Sahnish College, 220 8th Ave. E, New Town, ND 58763, USA
3 Agilent Technologies, 2850 Centerville Rd., Wilmington, DE 19808, USA
4 SCIEX, 1201 Radio Rd., Redwood City, CA 94065, USA
5 North Dakota Office of the Attorney General, Crime Laboratory Division, 2641 E Main Ave., Bismarck, ND 58501, USA
* Correspondence: evguenii.kozliak@und.edu (E.I.K.); alena.kubatova@und.edu (A.K.)

**List of Figures and Tables**

Table S1. Calibration data for PS, PMMA standards obtained for analytical SEC........................................2

Figure S1. Calibration curve for PS, PMMA standards obtained for analytical SEC separation ...................2

Figure S2. SEC preparative and analytical chromatograms for preliminary fractionation experiments ......3

Table S2. MW characterization of the five fractions obtained by preparative SEC and unfractionated alkali lignin conducted with ESI-TOF HR MS. .............................................................................................6

Figure S3. ESI-TOF HR MS spectra for SEC lignin fractions 1 – 5 ................................................................7

Figure S4. Aggregated TD-Py-GC-MS peak areas based on the GC-MS TIC response .................................9

Figure S5. TD-Py-GC-MS profiles for each SEC fraction based on the TIC peak areas response ...............10

Figure S6. $^{31}$P NMR spectra of SEC weight fractionated lignin samples.....................................................11

Figure S7. Relative abundance of characteristic functional groups determined by $^{31}$P NMR ....................12

Table S1. Calibration data for PS, PMMA standards obtained for analytical SEC

| TYPE | MW | log MW | tr, min |
|---|---|---|---|
| PMMA | 550 | 2.740363 | 9.783 |
| PMMA | 960 | 2.982271 | 9.404 |
| PMMA | 1780 | 3.25042 | 8.739 |
| PMMA | 2800 | 3.447158 | 8.401 |
| PMMA | 4640 | 3.666518 | 8.019 |
| PMMA | 6850 | 3.835691 | 7.710 |
| PMMA | 10280 | 4.011993 | 7.338 |
| PMMA | 17810 | 4.250664 | 6.856 |
| PMMA | 26080 | 4.416308 | 6.550 |
| PS | 580 | 2.763428 | 9.675 |
| PS | 1480 | 3.170262 | 8.879 |
| PS | 2340 | 3.369216 | 8.496 |
| PS | 5030 | 3.701568 | 7.860 |
| PS | 8450 | 3.926857 | 7.395 |
| PS | 19760 | 4.295787 | 6.655 |

Figure S1. Calibration curve for PS, PMMA standards obtained for analytical SEC separation

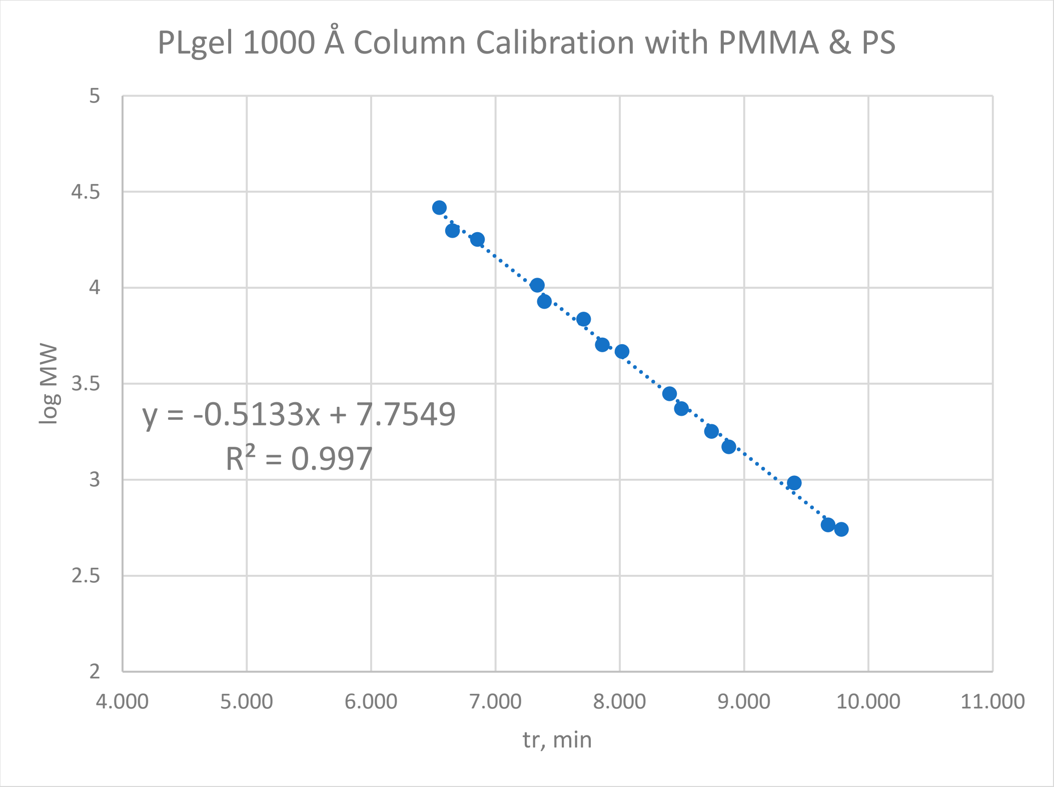

Figure S2. SEC preparative and analytical chromatograms for preliminary fractionation experiments

a) Preparation SEC - Fractionation 1 – focus on specific MW (not equal time intervals)

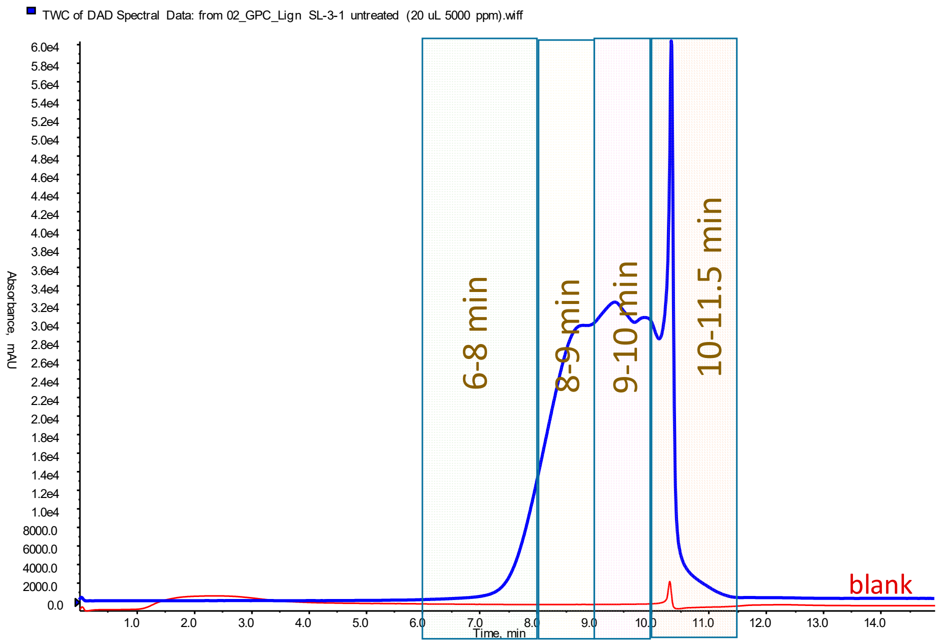


b) Analytical SEC - Fractionation 1 – focus on specific MW (not equal time intervals)

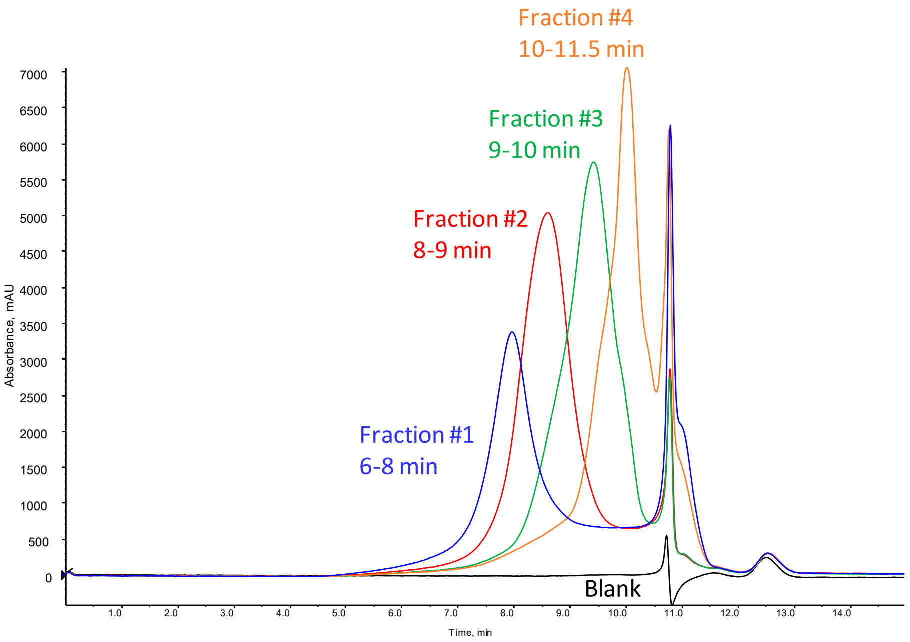

c) Preparation SEC - Fractionation 2 – focus on specific MW (not equal time intervals)

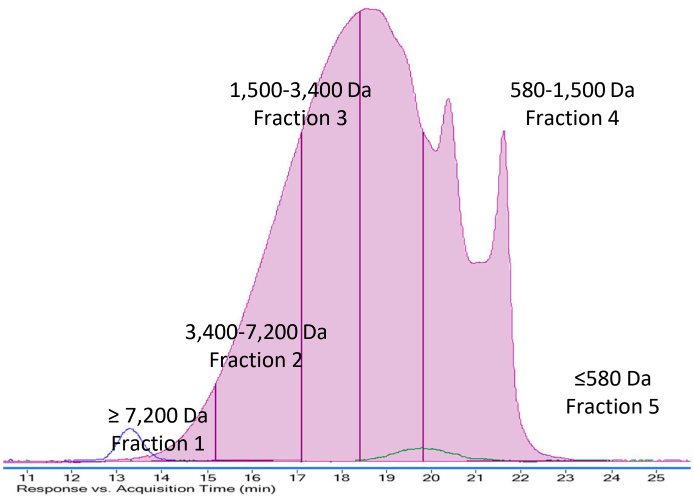


d) Analytical SEC - Fractionation 2 – focus on specific MW (not equal time intervals)

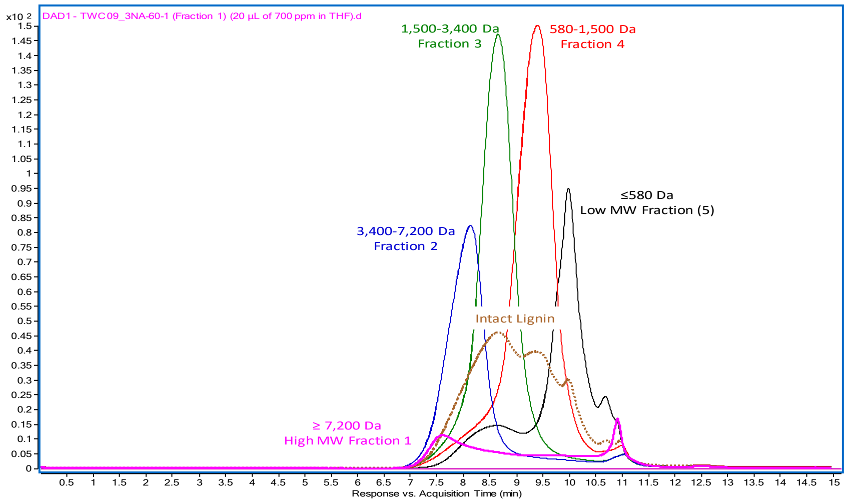

e) Preparation SEC - Fractionation 3– including pre and post-eluate

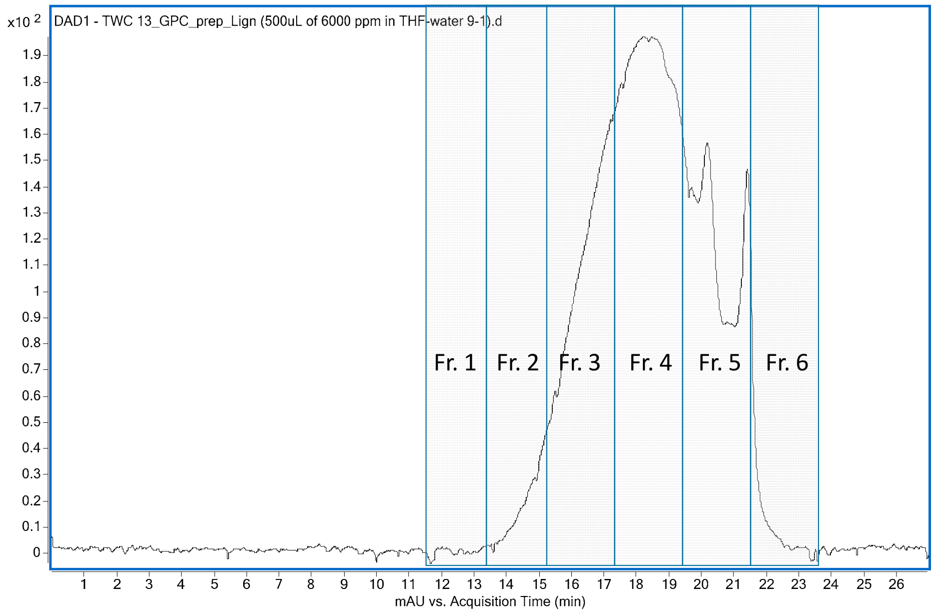


f) Analytical SEC - Fractionation 3 – – including pre and post-eluate

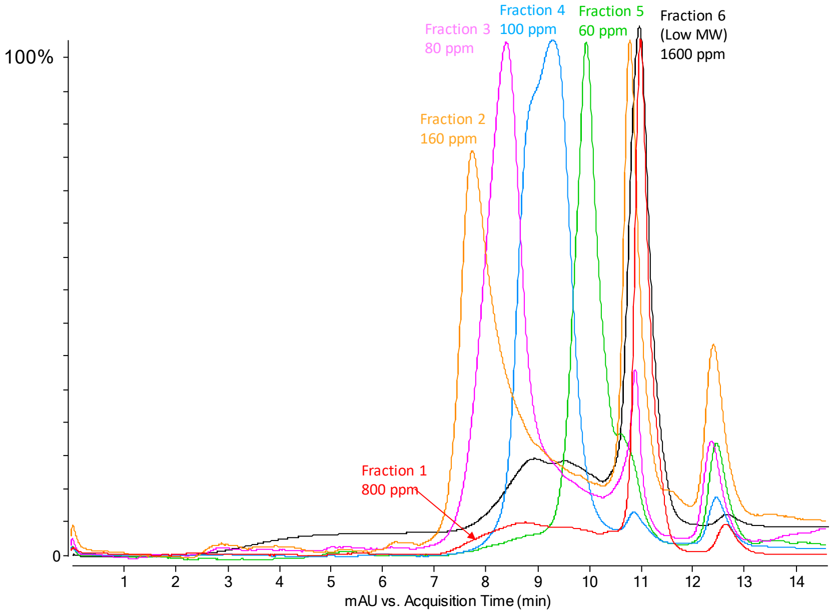

Table S2. MW characterization of the five fractions obtained by preparative SEC and unfractionated alkali lignin conducted with ESI-TOF HR MS.

| ESI-TOF HR TOF MS | | | | | | |
|---|---|---|---|---|---|---|
| **Fractions** | **1** | **2** | **3** | **4** | **5** | **Alkali Lignin** |
| **$M_n$** | 1330 | 1634 | 1028 | 1188 | 522 | 869 |
| **$M_w$** | 3160 | 3547 | 2266 | 2853 | 1335 | 1881 |
| **$M_z$** | 5261 | 5557 | 4500 | 4820 | 3321 | 3767 |
| **PDI** | 2.4 | 2.2 | 2.2 | 2.4 | 2.6 | 2.2 |

Figure S3. ESI-TOF HR MS spectra for SEC lignin fractions 1 – 5

MS data shown as the direct acquisition (m/z) and upon deconvolution (Da). All fractions were diluted 5 times except fraction 1, which was diluted 2 times.

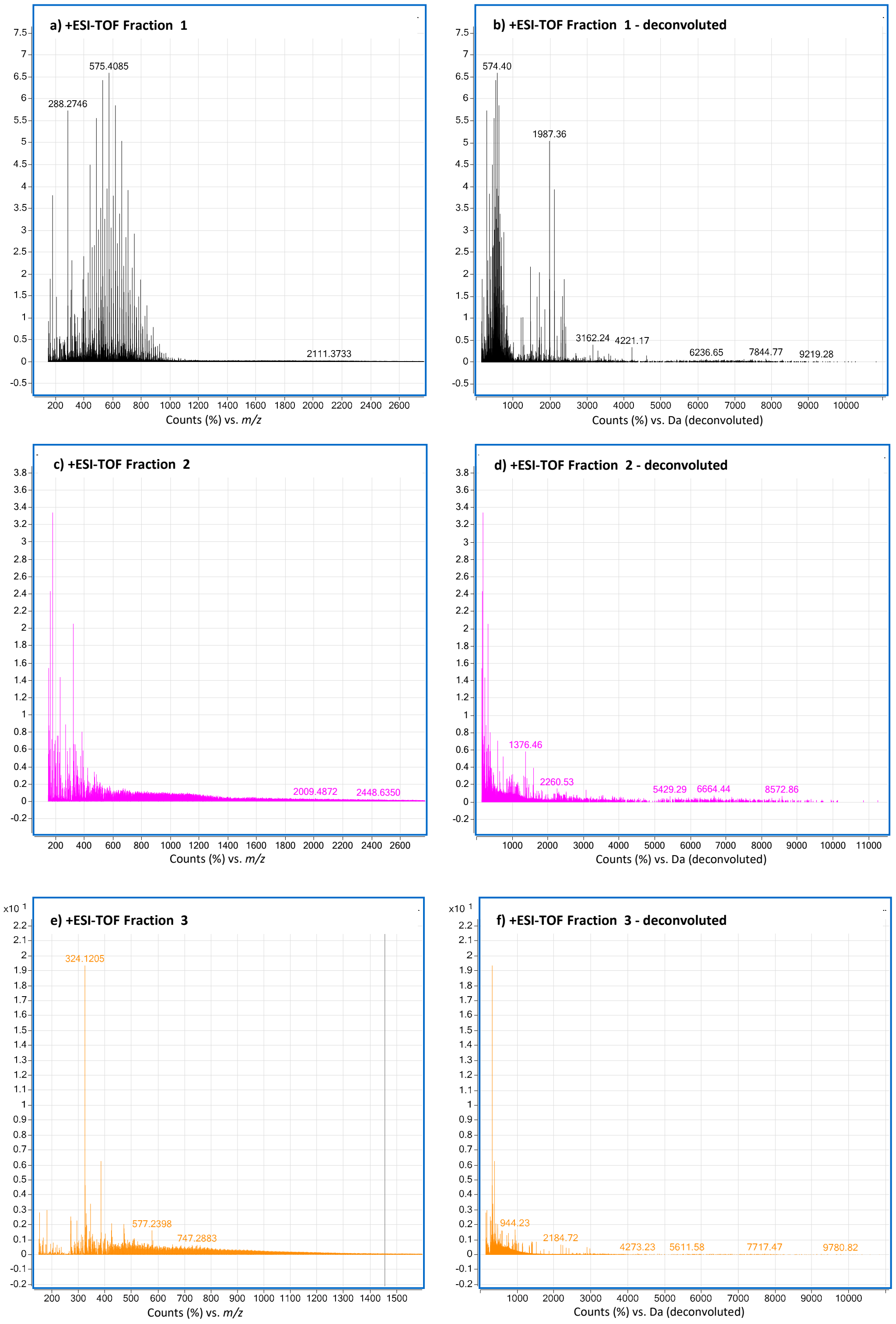

Figure S3 count.

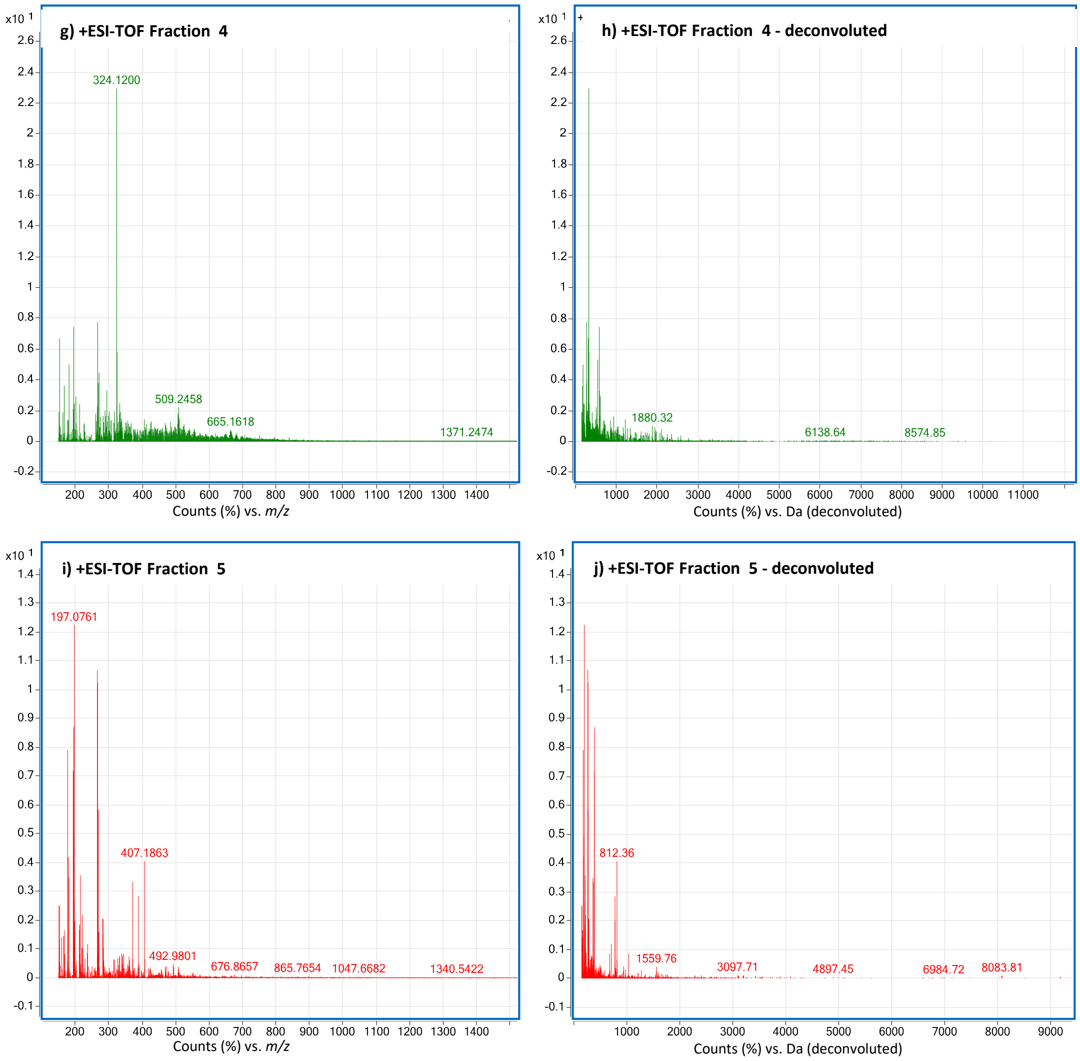

Figure S4. Aggregated TD-Py-GC-MS peak areas based on the GC-MS TIC response

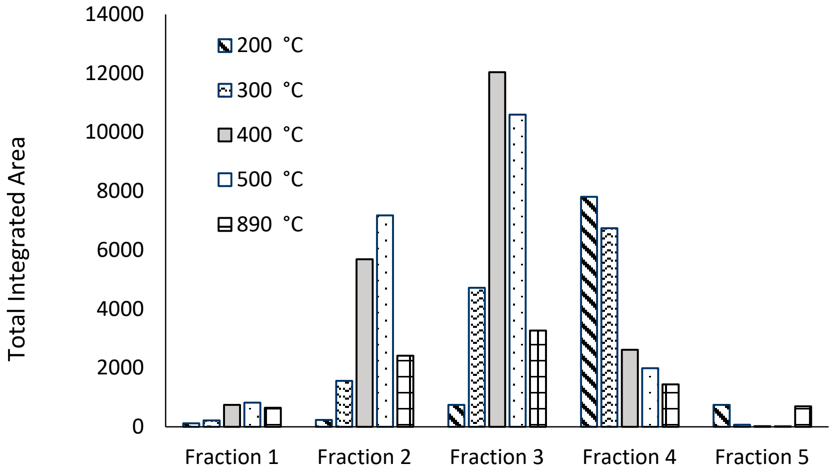

Figure S5. TD-Py-GC-MS profiles for each SEC fraction based on the TIC peak areas response

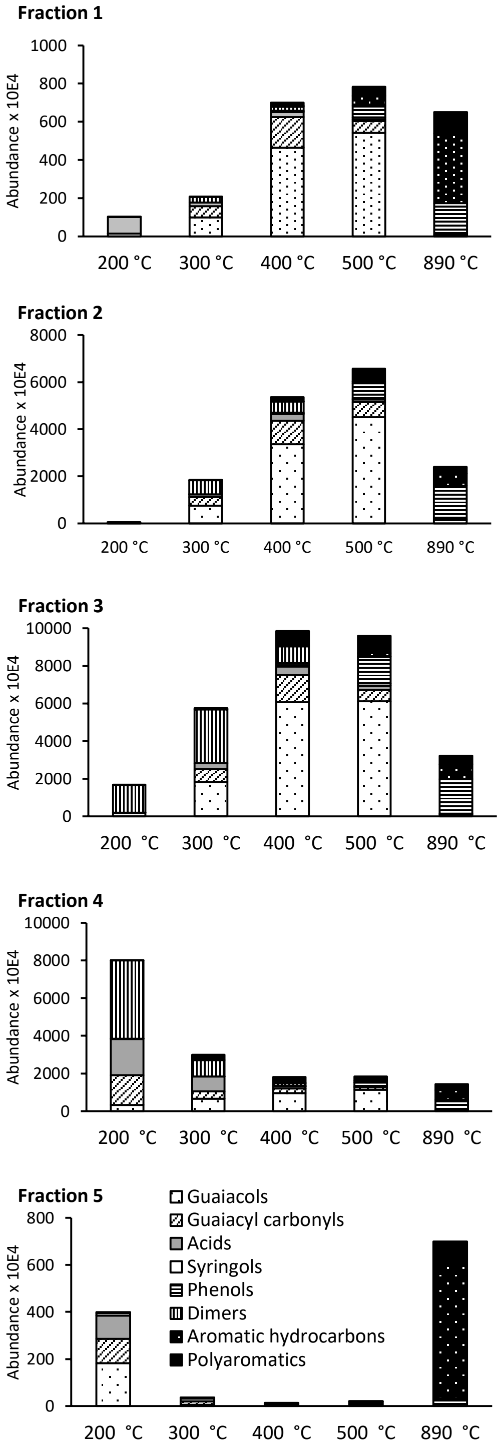

Figure S6. $^{31}P$ NMR spectra of SEC weight fractionated lignin samples.

The top spectrum belongs to the lowest molecular weight fraction, 3NA-81-6, while the second spectrum from the bottom is for the highest molecular weight fraction, 3-NA-2. The bottom spectrum is a pre-eluate fraction comprised mostly of impurities of carbohydrate origins.

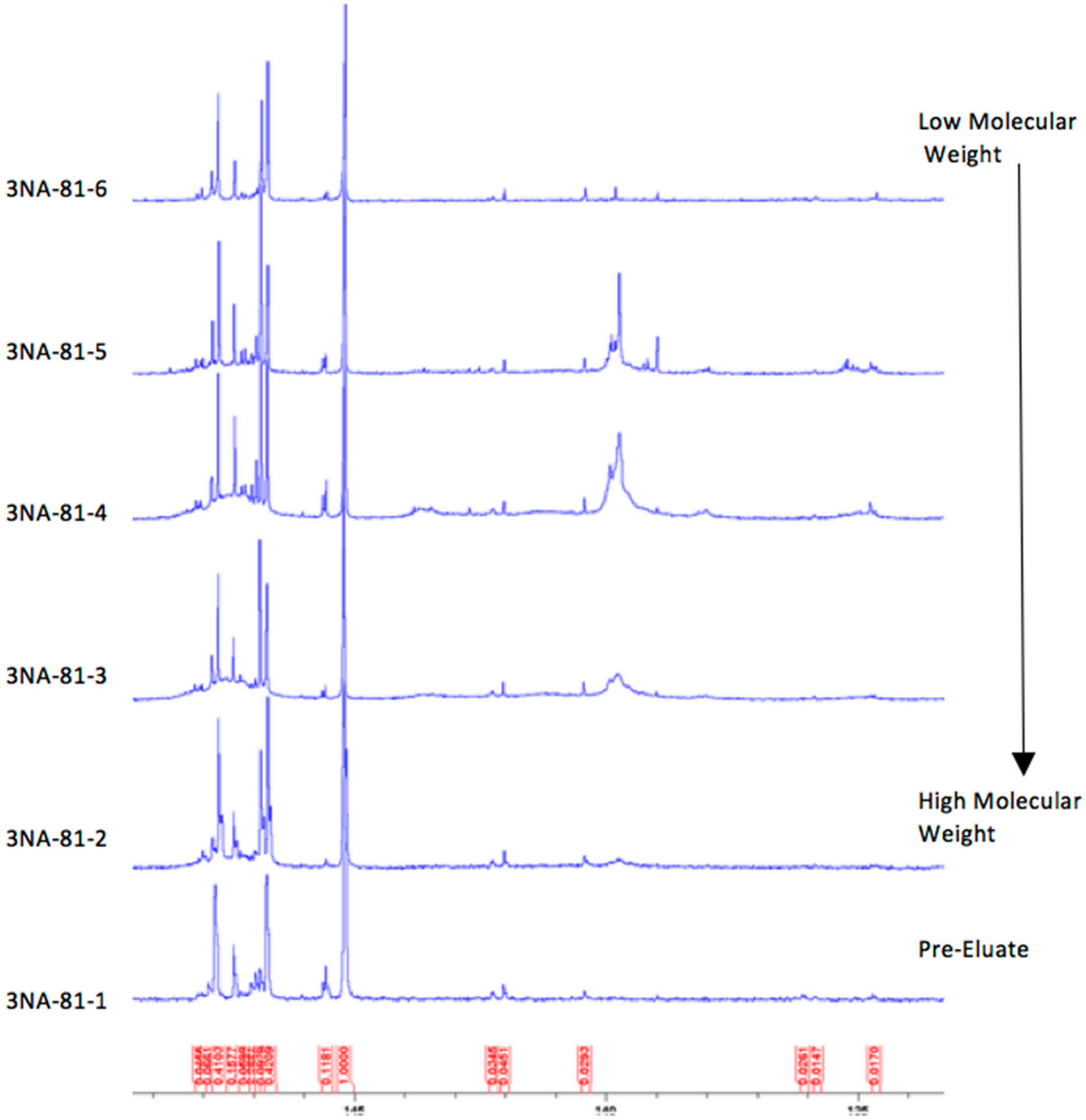

Figure S7. Relative abundance of characteristic functional groups determined by $^{31}$P NMR

a) Relative abundance

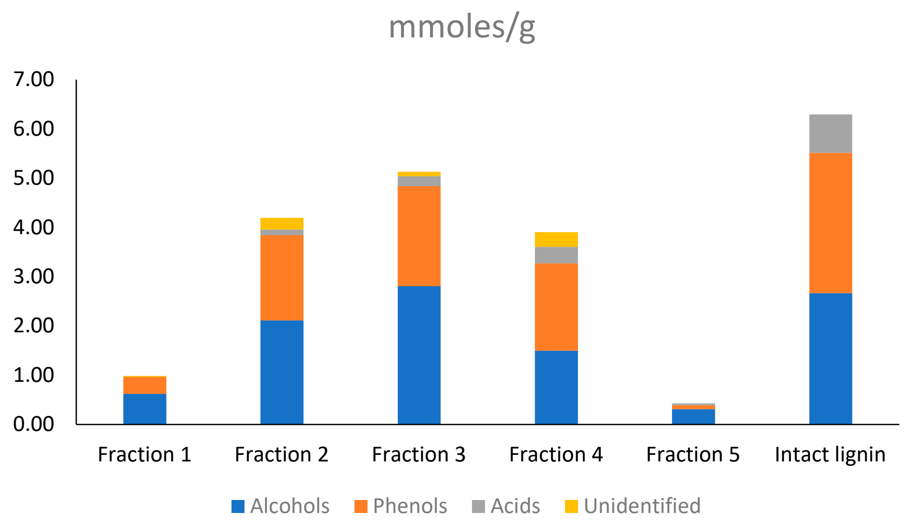


b) Normalized

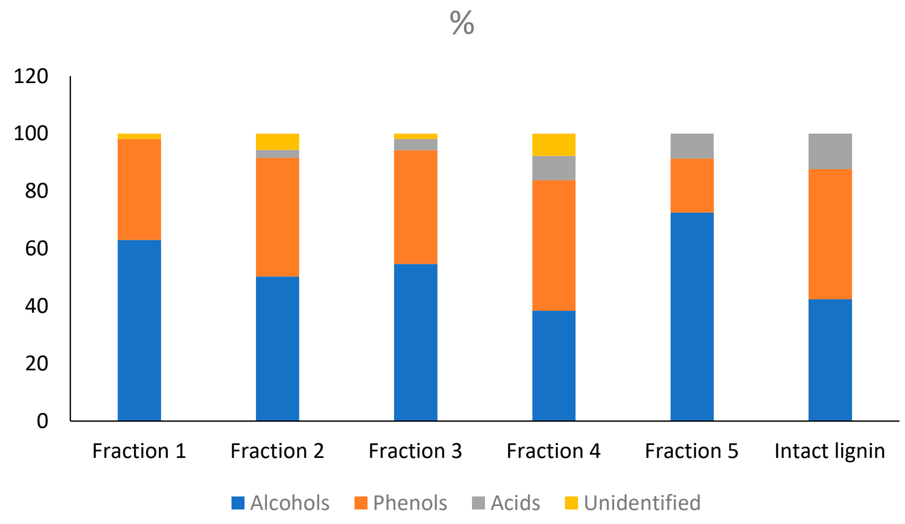

Supplement: Supplementary file 1 [file polymers-15-03956-s001.zip › polymers-2623485-supplementary.pdf]
